# Bilateral Terrien’s Marginal Degeneration and Posterior Polymorphous Dystrophy in a Patient with Rheumatoid Arthritis

**Published:** 2012-01

**Authors:** Siamak Zarei-Ghanavati, Mohammad-Ali Javadi, Shahin Yazdani

**Affiliations:** 1 Khatam-al-Anbia Eye Research Center, Mashhad University of Medical Sciences, Mashhad, Iran; 2 Ophthalmic Research Center, Shahid Beheshti University of Medical Sciences, Tehran, Iran

**Keywords:** Rheumatoid Arthritis, Corneal Ectasia, Posterior Polymorphous Dystrophy, Terrien’s Marginal Degeneration

## Abstract

**Purpose:**

To report an interesting case of concomitant bilateral Terrien’s marginal degeneration-like corneal ectasia and posterior polymorphous corneal dystrophy in a young man with quiescent rheumatoid arthritis.

**Case Report:**

A 24-year-old man with history of rheumatoid arthritis presented with bilateral decreased vision since four years ago. Slit lamp examination revealed bilateral circumferential peripheral corneal thinning and bulging with vascularization and lipid deposition in addition to band-like lesions in descemet’s membrane. Previous records revealed no gross corneal abnormalities up to 4 years ago. Corneal lesions were compatible with bilateral circumferential Terrien’s marginal degeneration concomitant with posterior polymorphous dystrophy.

**Conclusion:**

To our knowledge, this is the first report of concomitant bilateral Terrien’s marginal degeneration with peripheral corneal ectasia and posterior polymorphous dystrophy in a patient with rheumatoid arthritis. Bilateral circumferential involvement, younger age at presentation and total peripheral corneal ectasia as observed in this case are not typical for classic Terrien’s marginal degeneration.

## INTRODUCTION

Rheumatoid arthritis affects about 1% of the world’s population with predominant female involvement.^1^ Extra-articular manifestations may occur in almost any organ with variable incidence in different series. Common ocular presentations include Sjorgen’s syndrome, episcleritis, scleritis and keratitis.^2^–^6^ In cases with keratitis, arthritis is often inactive and patients demonstrate inactive systemic disease; this is in contrast to systemic associations of scleritis.^7^,^8^

Herein, we report an interesting case of bilateral Terrien’s marginal degeneration-like corneal ectasia and posterior polymorphous corneal dystrophy in a young man with quiescent rheumatoid arthritis. To the best of our knowledge, these manifestations have not previously been reported in the literature.

## CASE REPORT

**Figure 1.**
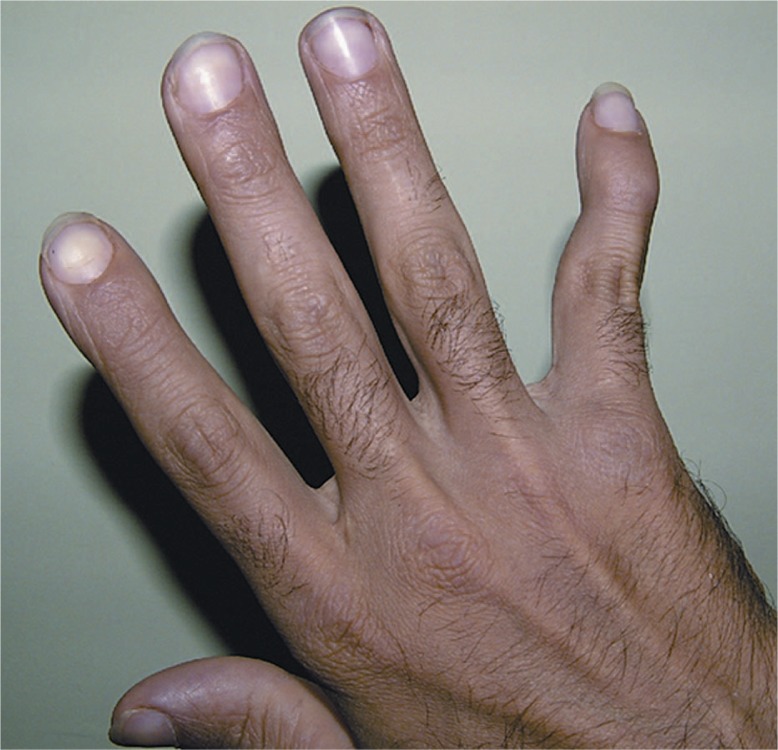
Swan-neck deformity in the fifth finger due to previous attacks of rheumatoid arthritis.

A 24-year-old man was referred to our center complaining of bilateral ocular irritation and progressive visual deterioration over the past 4 years. Past ocular history was negative for wearing contact lenses or previous ocular infections and there was no family history of ocular disorders. Previous records revealed no gross ocular abnormalities until 4 years ago. The patient reported pain, redness and swelling in the distal joints of his hands, and had been diagnosed with seropositive rheumatoid arthritis since the age of sixteen. The proximal interphalangial (PIP) and metacarpophalangial (MCP) joints in both hands were principally involved resulting in significant deformity in the fifth fingers (Fig. 1). At presentation, rheumatoid arthritis was well controlled with hydroxychloroquine under observation of a rheumatologist.

On ophthalmic examination, uncorrected visual acuity (UCVA) was 20/50 bilaterally which improved to 20/20 in both eyes with − 0.25 − 4.25@48 ° and − 0.75 − 2.00@100 ° in the right and left eyes respectively. Keratometric readings were 47.00×140, 43.25×50; and 43.00×100, 44.75×10 in the right and left eyes, respectively.

**Figure 2.**
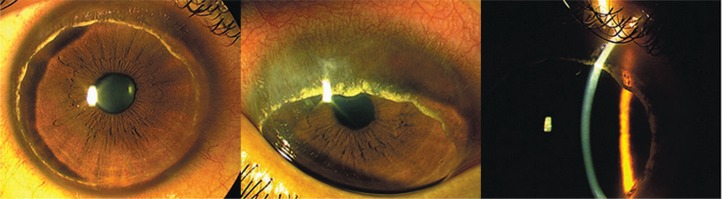
Circumferential corneal involvement (left image) in a 24-year-old man with rheumatoid arthritis. Note the peripheral corneal thinning, vascularization and lipid deposition (middle image); and significant peripheral thinning and ectasia with slit beam illumination (right image).

Slit lamp biomicroscopy revealed normal lid margins without any signs of meibomian gland dysfunction or blepharitis; the tear meniscus and tear break up time were within normal limits. The conjunctiva was slightly injected near the limbus but the sclera was normal. Marked corneal thinning and bulging was noted in the whole periphery of the cornea; this circumferential gutter was 1.5–2 millimeters in width with superficial vascularization but had an intact epithelium. Circumferential lipid deposition was detected in front of the leading vascular edge. The central cornea however, was clear and free of lipid deposits and vascularization (Fig. 2). These features were similar to manifestations of Terrien’s marginal degeneration.

**Figure 3.**
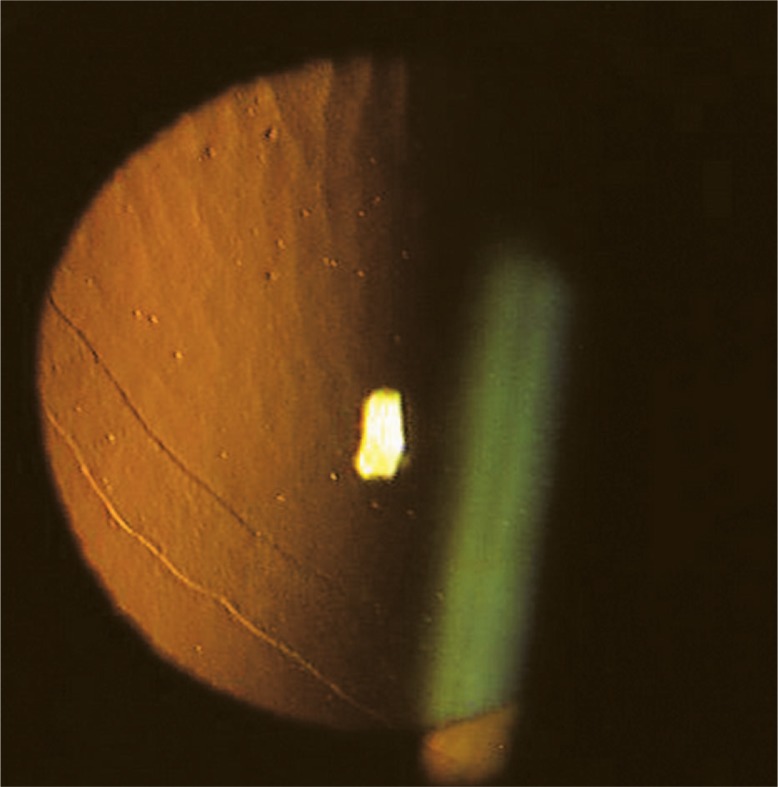
Slit lamp photography with retro-illumination reveals band-like lesions together with dispersed vesicles compatible with posterior polymorphous dystrophy.

In addition to the above-mentioned findings, broad bands and vesicles were present on the corneal endothelial surface. The band-like lesions had parallel scalloped edges which did not taper toward the ends and were more prominent with retroillumination (Fig. 3). The appearance of the lesions was typical for posterior polymorphous corneal dystrophy.

The anterior chamber was deep and the lens and iris were normal with no sign of current or previous inflammation. Intraocular pressure measured with Goldmann applanation tonometry was 6 and 7 mmHg in the right and left eyes, respectively. Gonioscopy was unremarkable and the angle appeared open. Posterior segment examination was also unremarkable. Ultrasonic pachymetry revealed central and mean peripheral thickness of 450 μm and 390 μm respectively in the right cornea. Corresponding values for the left cornea were 506 μm and 250 μm. Corneal thickness was most significantly attenuated in the superior corneal periphery.

## DISCUSSION

Prominent clinical features in the patient reported herein included peripheral corneal gutter with superficial vascularization, an intact epithelium and lipid deposition in front of the leading vascular edge consistent with a diagnosis of Terrien’s marginal degeneration.

Terrien’s marginal degeneration is a rare, slowly progressive peripheral corneal thinning disorder first described in 1900.^9^ The condition is usually bilateral and starts in the superonasal quadrant as fine, yellow-white punctate stromal opacities that may progress along the circumference of the cornea. It is further characterized by vascularization at the base of the lesions, and scarring and lipid infiltration at its leading edge. Although the etiology of this entity remains unknown, its clinical characteristics and course have been well described. The classic form most commonly affects patients older than 40 years of age without rheumatoid disease and shows extremely slow progression.^10^–^12^ Austin and Brown^13^ have described an inflammatory variant of Terrien’s marginal degeneration in younger subjects with no history of rheumatoid disease; this is characterized by recurrent and disabling attacks of acute pain and inflammation.

The case reported herein had early onset circumferential corneal involvement, distinguishing it from typical Terrien’s marginal degeneration which is usually slowly progressive and involves the superior cornea in patients older than 40 years of age. Although our patient presented at young age, bilateral circumferential involvement with minimal symptoms differentiates it from inflammatory Terrien’s marginal degeneration which is characterized by severe pain and episcleritis or superficial scleritis in the majority of patients. Furthermore, corneal involvement has been reported to be localized in inflammatory forms, as opposed to our case.^13^ Central corneal thinning (in the right eye) and total peripheral corneal ectasia in our patient were also atypical for Terrien’s marginal degeneration,^14^ although Singh and Malik^15^ reported an interesting case of bilateral total corneal ectasia due to Terrien’s marginal degeneration in a patient with no rheumatologic illness.

Concomitant rheumatoid arthritis and Terrien’s marginal degeneration is very rare with only a single report back in 1977 by Awan^16^ who described bilateral Terrien’s marginal degeneration in a patient with rheumatoid arthritis. The reported patient had severe conjunctival shrinkage resembling ocular pemphigoid. In less than two years, the right eye was lost due to spontaneous corneal perforation and the left one lost sight because of conjunctival scarring and shrinkage.

Ocular involvement in rheumatoid arthritis tends to occur in more advanced stages of systemic disease. Corneal involvement can be central or peripheral; the latter is more frequent because of closer access of inflammatory cells.^17^ These manifestations are usually not severe; however, a small percentage of patients with rheumatoid arthritis present with severe ocular involvement which may lead to corneal perforation. Corneal melting in rheumatoid arthritis is usually minimally painful and the eye tends to be quiet.^7^,^8^ Our patient had an eight-year history of rheumatoid arthritis; when the patient was referred to us, the disease was well controlled but hand deformity documented previous attacks.

It is possible that the corneal changes described herein may represent unrecognized manifestations of peripheral keratolysis associated with rheumatoid arthritis; alternatively our patient may simply be a case of concomitant Terrien’s marginal degeneration in a patient with rheumatoid arthritis. If we consider the latter hypothesis, the current report and a previous one describing corneal perforation^16^ may suggest a possible role for rheumatoid arthritis as an aggravating factor in severe and atypical presentations of Terrien’s marginal degeneration.

To our knowledge, this is the first report of bilateral Terrien’s marginal degeneration-like corneal ectasia concomitant with posterior polymorphous dystrophy in a patient with rheumatoid arthritis. Younger age at presentation and bilateral circumferential peripheral corneal ectasia in this case differentiate it from classic Terrien’s marginal degeneration.
